# The reliability of side to side measurements of upper extremity activity levels in healthy subjects

**DOI:** 10.1186/1471-2474-11-168

**Published:** 2010-07-22

**Authors:** Miguel Acuna, Tal Amasay, Andrew R Karduna

**Affiliations:** 1 Departments of Biological Sciences and Kinesiology, Chico State University, Chico, CA 95929, USA; 2 Department of Sport & Exercise Sciences, Barry University, Miami Shores, FL 33161, USA; 3 Department of Human Physiology, University of Oregon, Eugene, Oregon 97403, USA

## Abstract

**Background:**

In both clinical and occupational settings, ambulatory sensors are becoming common for assessing all day measurements of arm motion. In order for the motion of a healthy, contralateral side to be used as a control for the involved side, the inherent side to side differences in arm usage must be minimal. The goal of the present study was to determine the reliability of side to side measurements of upper extremity activity levels in healthy subjects.

**Methods:**

Thirty two subjects with no upper extremity pathologies were studied. Each subject wore a triaxial accelerometer on both arms for three and a half hours. Motion was assessed using parameters previously reported in the literature. Side to side differences were compared with the intraclass correlation coefficient, standard error of the mean, minimal detectable change scores and a projected sample size analysis.

**Results:**

The variables were ranked based on their percentage of minimal detectable change scores and sample sizes needed for paired t-tests. The order of these rankings was found to be identical and the top ranked parameters were activity counts per hour (MDC% = 9.5, n = 5), jerk time (MDC% = 15.8, n = 8) and percent time above 30 degrees (MDC% = 34.7, n = 9).

**Conclusions:**

In general, the mean activity levels during daily activities were very similar between dominant and non-dominant arms. Specifically, activity counts per hour, jerk time, and percent time above 30 degrees were found to be the variables most likely to reveal significant difference or changes in both individuals and groups of subjects. The use of ambulatory measurements of upper extremity activity has very broad uses for occupational assessments, musculoskeletal injuries of the shoulder, elbow, wrist and hand as well as neurological pathologies.

## Background

When assessing upper extremity function, clinicians are generally limited to the use of measurements that can be made during an office visit (eg, physical exams, range of motion, self report assessments). However, as suggested by Zhou et al., these "traditional methods lack objective standardized analyses for evaluating a patient's performance and assessment of therapy effectiveness." [1] While evaluations in a clinical setting provide important information about a patient's capacity for performing common activities of daily living, they do not provide information about upper extremity activity outside of the clinic, both at home and in an occupational setting.

There are many commercially available systems that utilize accelerometers for assessing ambulatory measurements of physical activity. These systems convert acceleration data to arbitrary units of movement "counts" using threshold crossings, maximum values or integration algorithms [2]. Although originally developed for placement on the trunk to serve as surrogate measurements of energy expenditure, this methodology has been adapted for assessing movement of the upper extremity. For this approach, the activity of the pathological side is generally compared to that of the uninvolved side. This has been used in patients with stroke [3-5] and complex regional pain syndrome [6]. Another approach for assessing arm motion is to place a sensor on the humerus for direct assessment of arm elevation. Older studies have secured pressure transducers, [7] liquid level sensors, [8] or mercury microswitches [9,10] to the arm. However, more recent studies have used linear accelerometers as tilt sensors for both ergonomic assessments [11-14] as well as assessing arm position during the course of daily activities [15].

Regardless of where the sensor is placed and what analysis is conducted, if a researcher wishes to use the contralateral upper extremity as a control, it is important to know to extent to which there are inherent side to side difference in daily arm usage. Previous studies have demonstrated that there is preferential use of the dominant hand in complex tasks, regardless of biomechanical efficiency [16,17]. Additionally, laboratory studies have documented side to side differences in shoulder range of motion, [18] electromyography [19] and neural control of movement [20]. However, recent studies by Coley et al. suggest that all day measurements of arm motion are not significantly different between dominant and non-dominant sides [15,21].

Rather than looking at whether or not there are significant differences between sides in healthy subjects, our goal is to determine the reliability and variability of these measurements. From this analysis, we can determine the feasibility of detecting: 1) significant side to side differences in individual patients, 2) significant side to side differences in groups of patients and 3) significant group differences between patients and control subjects.

## Methods

### Subjects

Thirty two healthy individuals (16 males, 16 females) with a mean age of 23 (+/- 7) years, a mean body mass of 70 (+/- 14) kg and a mean height of 170 (+/- 9) cm agreed to participate in the study. According to the Edinburgh Handedness Inventory, [22] thirty one subjects were classified as being right handed and one was left handed. Subjects were asked about the health of their shoulders and any subject that reported a past or present shoulder injury was excluded. In general, subjects were healthy college students. Prior to testing, all subjects read and signed an informed consent form approved by the Institutional Review Board at the University of Oregon.

### Instrumentation

The Virtual Corset [VC] (Microstrain Inc. VT, USA), which is a tri-axial linear accelerometer, was used to record ambulatory measurements. The device is battery operated, functions without cables, contains a 2 Mb data logger with a 7.6 Hz collecting rate and 4.9 hour recording capability, has a mass of 72 grams and measures 6.8 cm × 4.8 cm × 1.8 cm. The VC was secured with double sided industrial tape and self-adhesive wrap.

### Protocol

**Figure 1:**
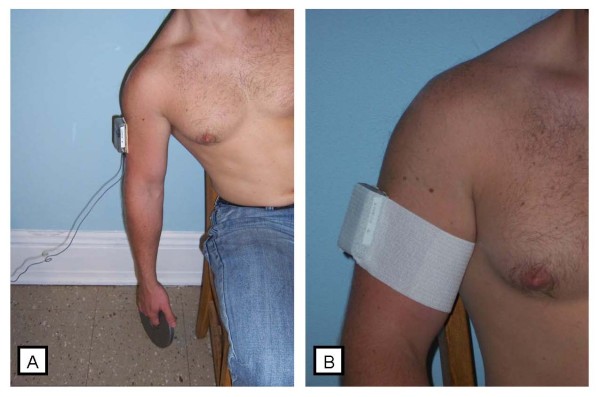
**Placement of Virtual Corset**. (A) The Virtual Corset was set as close to zero orientation as possible when the arm was hanging at the side of the body with the subject holding a 1 kg mass. (B) The Virtual Corset was further secured with self-adhesive wrap

Two VC's were placed on each subject - one on each arm. Prior to placement of the VC's, the subject was instructed to maintain a seated upright posture while holding a 1 kg mass in one hand. Direction was given to perform lateral trunk flexion towards this arm; thus resulting in the hanging arm being roughly oriented with the line of gravity. The VC was attached on the lateral side of the humerus, via double sided tape, with the superior end just proximal to the deltoid tuberosity (figure 1A). The VC was connected to a computer, so real time feedback of the orientation of the sensor was possible. The VC was secured to the arm with the orientation as close to zero degrees as possible, as determined by the on-line feedback. After the application of the VC the area of skin surrounding it was outlined for observation of possible displacement. The subject was then allowed to relax to a comfortable posture where the device was further secured with self-adhesive wrap (figure 1B). Instruction was then given to return to the "hanging arm" position for collection of the zero gravity data, which was used as a means of determining any misalignment of the VC [23]. The subject held the position while two seconds of data were collected.

The VC was then disconnected from the computer and the process was repeated with another VC for the other arm. The subject then left the lab and returned approximately four hours later. Upon returning, the sensors were removed and the accelerometer data were downloaded to a computer. No subject reported any concerns about the sensors impacting their activities or with the sensors slipping. These VC's had previously been upgraded by the manufacturer to allow for collection of the raw accelerometer data, rather than the standard lateral bending and flexion extension bending angles described in the manual. A previous validation study has demonstrated a static accuracy of approximately 1 degree and a dynamic accuracy of approximately 3 degrees [24].

### Data Analysis

Given that the z-axis is aligned with the long axis of the arm, elevation angles were calculated as a function of all three accelerometer data coordinates as follows:(1)

where x, y and z are the outputs (in g's) from the triaxial accelerometer [24]. The zero gravity data for each VC was calculated as the average data over the two seconds of data collection. Due to some early returns to the lab and to ensure consistency between subjects, only the first 3.5 hours of data collect were analyzed for each subject.

The elevation data were analyzed with all previous approaches found in the literature: percent time above a given angle (30, 60 and 90 degrees); [10] cumulative probability function, which represents the 10^th^, 50^th ^and 90^th ^percentiles of elevation data; [8,23] weighted score, which takes into account the amount of arm elevation as well as the time spent at each angle; [15] and jerk time [25]. Note that the first parameter represents the percent time of their day spent between that angle (eg 30 degrees) and 180 degrees (max elevation angle). In order to calculate the jerk time, the entire data set was run through an algorithm that looked for the number of adjacent data points contained within a single 10 degree bin (eg, 0-10, 10-20, etc.). If the number of points within that bin represented a time of less than one second, the points were classified as dynamic; otherwise they were classified as static [25]. The jerk time was defined as the number of dynamic points divided by the total number of points.

An additional parameter was calculated from the acceleration data: activity counts [2]. Activity counts were calculated with an algorithm adopted from that used by the Actical Physical Activity Monitor (Philips Respironics, Bend, Oregon). First the magnitude of the resultant acceleration vector was calculated using data from all three accelerometers (if the sensor was not moving, this magnitude would always be equal to 1 g, no matter what the orientation of the sensor). The resultant data were then run through a 2^nd ^order band pass Butterworth filter (0.5 - 3 Hz), rectified and finally integrated over the entire trial to calculate the area under the acceleration (g) - time (sec) curve.

### Statistical Analysis

We were interested in examining the feasibility of three assessments with our proposed measurements: 1) side to side differences in individual patients, 2) side to side differences in groups of patients and 3) group differences in patients and control subjects. The first step was to assess measurement reliability with the use of an ICC (3,1) analysis for each parameter. The ICC (3,1) model was selected because there was only one rater and this rater was not selected from a larger group of raters. Standard error of the measurement (SEM) values were calculated as the square root of the error variance (mean square error from the Analysis of Variance used to calculate the ICC) [26,27]. In order to examine the extent to which a side to side difference in an *individual *patient should be considered more that due to inherent side to side variability (goal 1), we calculated the minimal detectable change scores (MDC) at the 95% confidence level as follows [27]:(2)

Note that in order to compare MDC scores between all the parameters in the present study, the values were also normalized to the overall mean for that particular parameter (MDC%) [28]. Normalizing to the mean is appropriate since all of our variables are ratio measurements. In order to explore the ability to detect *group *differences, we ran a sample size analysis for each parameter with the following assumptions: power = 80%, alpha = 5%, minimal detectable difference = 10% of the overall mean value for that particular parameter. The sample size estimate was done twice - once assuming paired samples (goal 2 - side to side differences for the same subjects) and once assuming unpaired samples (goal 3 - comparison between groups). Analyses were run with SPSS version 17 (SPSS, Chicago, Illinois) and G*Power 3.1.0 [29]. In SPSS, the ICC (3,1) is represented by a two-way mixed single measures analysis.

## Results

The mean (and standard deviation) of the zero gravity position was 3°(+/- 2°). As the threshold for percent time above a given angle was increased, the percent time dramatically dropped, with a tenfold decrease from > 30 degrees to > 90 degrees. Similarly, for the percentiles, there was a dramatic decrease from the 90^th ^to the 10^th ^percentile. ICC and SEM values for all parameters are presented in table 1. In general, the means for the non-dominant side were similar to the dominant size. All ICC values were below 0.8 except for the activity counts per hour (0.990), jerk time (0.963) and percent time above 30 degrees (0.832).

**Table 1:** Reliability Data

Parameter	Dominant	Non-Dominant	Difference	ICC	SEM
Time > 30°[%]	48.0 (13.3)	45.8 (15.3)	2.2 (8.3)	0.832	5.9

> 60°[%]	12.7 (10.3)	13.9 (11.3)	-1.2 (8.5)	0.696	6.0

> 90°[%]	3.9 (4.5)	2.5 (3.2)	1.4 (3.4)	0.627	2.4

Percentiles 10^th ^[deg]	66.4 (20.7)	62.9 (15.5)	3.5 (15.6)	0.638	11.0

50^th ^[deg]	30.4 (9.1)	29.3 (11.1)	1.0 (7.2)	0.750	5.1

90^th ^[deg]	9.6 (5.2)	9.1 (2.5)	0.5 (2.8)	0.393	2.0

Jerk Time [%]	36.5 (10.6)	35.3 (10.7)	1.2 (2.9)	0.963	2.0

Weighted Score [unitless]	117.3 (39.9)	111.8 (40.9)	5.4 (30.1)	0.723	21.3

Activity Counts [count/hour]	117.1 (39.7)	112.6 (40.1)	4.5 (5.6)	0.990	3.9

Means (SD) for the dominant and non-dominant sides, well as the difference scores. Results also presented for the intraclass correlation coefficient [ICC (3,1)] and standard error of the measurement [SEM].

For both the individual and group analyzes, the variables were ranked based on their MDC percentages and sample sizes needed for the paired t-tests. The order of these rankings was found to be identical and is presented in table 2. As with the ICC values, the top ranked parameters were activity counts per hour (MDC% = 9.5, n = 5), jerk time (MDC% = 15.8, n = 8) and percent time above 30 degrees (MDC% = 34.7, n = 9). The sample size for independent samples was also calculated and ranged from 3 to 75 times larger than for the paired t-test calculations (table 2).

**Table 2:** Minimal detectable change scores and projected subject numbers

Parameter	MDC	%MDC	n for paired t-test	n for independent t-test
Activity Counts [count/hour]	10.9	9.5	5	376

Jerk Time [%]	5.7	15.8	8	274

Time > 30°[%]	16.3	34.7	9	292

50^th ^Percentile [deg]	14.1	47.2	47	352

10^th ^Percentile [deg]	30.5	47.2	47	260

Weighted Score [unitless]	59.0	51.5	57	376

90^th ^Percentile [deg]	5.5	58.4	75	232

Time > 60°[%]	16.6	124.5	309	2184

Time > 90°[%]	6.6	204.9	787	4908

Minimal detectable change scores, presented in the units of the parameter (MDC) and as a percentage of the overall mean of that parameter (%MDC). Also presented are the number of subjects required to detect a 10% difference for that parameter with an 80% power and an alpha level of 5% for paired and independent t-tests.

## Discussion

The goal of the present study was to examine the side to side differences in ambulatory recordings of arm usage in healthy subjects. This is important because a better understanding of inherent side to side differences will lead to a better understanding of side to side differences in patients or workers with upper extremity pathologies. The more reliable side to side measurements are, the easier it will be to detect differences between affected and non-affected sides in patients with pathologies. However, it is important to emphasize that we did not test any patients with shoulder pathologies in the current study.

Ambulatory measurements can be used in two fundamental ways - to examine changes in individual patients and changes or difference in groups. For individual patients, a clinician might ask the following question - "Is there a significant improvement in this score after a given treatment?" The first step in answering this question is to determine whether or not the change score is larger than can be reasonably accounted for by measurement error. The threshold for this determination is the MDC [27]. For example a recent study of cervical spine range of motion determined that the MDC was higher for extension (9 degrees) when compared to right lateral flexion (6 degrees) [30]. This would imply that a larger change in extension would have to be recorded in order for it to be considered a true change. For the present study, it is difficult to compare MDC values between all nine parameters because of differences in units (eg, counts/hour vs percentage). We overcame this limitation by normalizing the MDC scores to the overall means for each parameter. At one extreme, the %MDC for activity counts was 9.5%, while the %MDC for time greater than 90 degrees was 205%. It is important to note that changes above the MDC only indicate that the change was not due to measurement error, not whether it is clinically meaningful. For that, one would need to determine the minimal clinically important difference (MCID), [27] which is beyond the scope of the present study.

**Figure 2:**
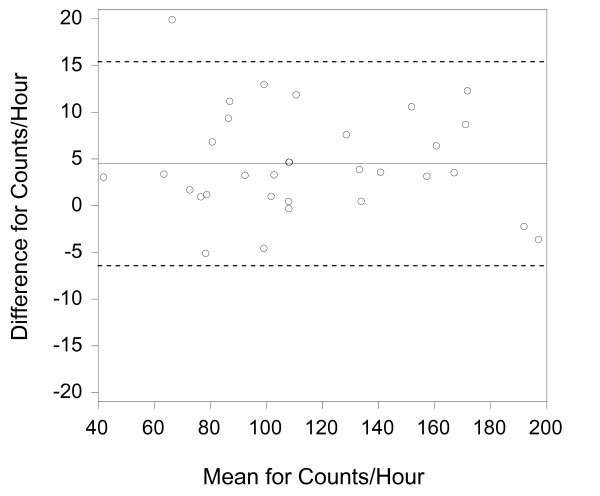
**Activity Counts Bland-Altman Graph**. The solid lines represent the mean difference (or bias) between the sides and the dashed lines represent the 95% limits of agreement. Analyses were conducted with Analyse it Software http://www.analyse-it.com

**Figure 3:**
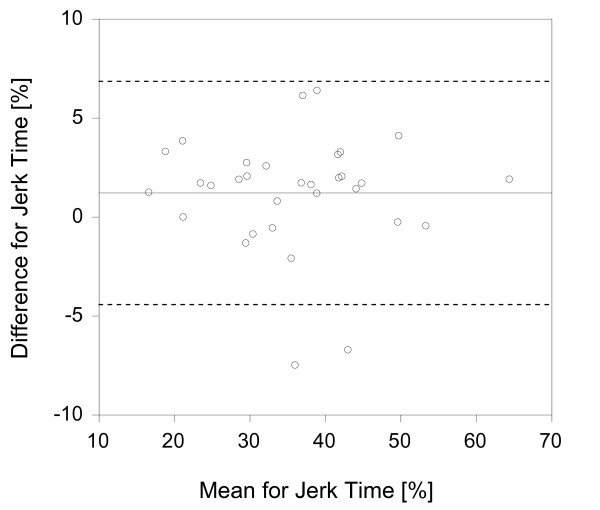
**Jerk Time Bland-Altman Graph**. The solid lines represent the mean difference (or bias) between the sides and the dashed lines represent the 95% limits of agreement. Analyses were conducted with Analyse it Software http://www.analyse-it.com

**Figure 4:**
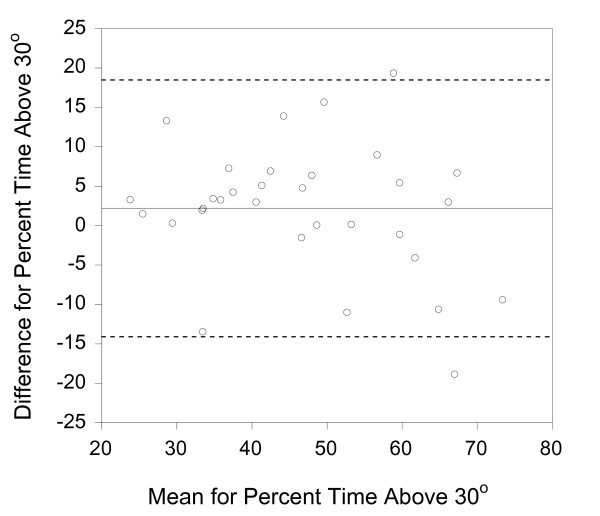
**Percent Time above 30 degrees Bland-Altman Graph**. The solid lines represent the mean difference (or bias) between the sides and the dashed lines represent the 95% limits of agreement. Analyses were conducted with Analyse it Software http://www.analyse-it.com

In order to provide a visual representation of the data, Bland-Altman graphs were created (figures 2, 3, 4) [31]. It should be noted that the distance between the 95% limits of agreement and the mean (or bias) is the same as the MDC values presented in table 2. There does not appear to be greater differences at higher mean values for any of the parameter. However, there is a slight positive bias for the activity counts.

For group differences, information from the present study can be used to demonstrate the number of subjects that would be needed to reveal a statistically significant difference. To detect a 10% difference in means (with alpha = 5% and power = 80%), the number of subjects varied greatly, from n = 5 for activity counts, up to n = 787 for percent time greater than 90 degrees. There appears to be a natural break after the top three parameters (activity counts per hour, jerk time, and percent time above 30 degrees), all of which required less than 10 subjects. These paired comparisons are more appropriate for situations in which a researcher hypothesizes a side to side difference due to a pathology, such as a stroke and in some instances Parkinson's disease [32]. However, in all cases, care should be taken in assuming impairments are unilateral. For example, in patients who have experienced a hemispheric stroke, there is evidence of motor deficits on the ipsilateral side [33-35]. Additionally, approximately 35% of patients who present with a painful full thickness rotator cuff tear on one side have been found to also have a full-thickness tear on the contralateral side [36]. In both of these cases, however, it would be of interest to know whether there is one side in which there is less arm usage.

If side to side comparisons are not possible, the next best option would be to compare the motion with healthy controls. However, as can be seen by the results in table 2, this would require a dramatic increase in the number of subjects required for all parameters. It should be noted that the subject numbers presented in the current study are meant as an example for comparison between parameters. The number of subjects for any given study would depend upon the expected difference in means, and alpha and power levels.

From a statistical point of view, for both individual and group comparisons, activity counts per hour, percent time above 30 degrees and jerk time appear to be the most appropriate parameters. Interestingly, it could also be argued that each of these parameters adds something different to the description of arm motion.

The first parameter, activity counts, provides a global assessment of arm motion, independent of any calculation of joint angle. This type of analysis has been used a great deal for both whole body analysis [2,37] as well as arm motion [3-6]. One of the biggest concerns with this approach is that different researchers, as well as commercially available systems, use different algorithms, so comparing between studies can be difficult.

The second parameter, percent time above a set elevation angle, can significantly differentiate workers with shoulder pathology and asymptomatic controls. Svendsen et al. [38] and Punnett et al. [39] both found that percent time about 90 degrees was the critical threshold, while Ohlsson et al. [40] found that time about 60 degrees was enough. Both of these parameters were found to be at the bottom of the ranking of parameters in table 2. However, these studies are related to the risk factor in an occupational setting and are more associated with what workers are expected to do, as opposed to what patients with a given pathology choose to do during their daily activities. While shoulder elevation above 30 degrees may not be a risk factor for the development of shoulder pathologies, it may be a better assessment of whether or not a patient who already has a pathology is using their involved arm during activities of daily living.

Finally, for the third parameter, jerk time, we adopted the approach of Moller et al. by creating 10 degree bins of arm elevation angles (0-10, 10-20, etc) and calculating the jerk time or "percentage of the cycle time spent in sequences shorter than 1 s within the same exposure category." [25] Jerk time is believed to be an assessment of the repetitiveness of a task, [41] and is probably closely related to the assessment of angular velocity reported by Coley et al. [21] Clearly, the size of these bins and sequence times are somewhat arbitrary and could be adjusted depending on the application. A more comprehensive approach involves performing an Exposure Variation Analysis (EVA), in which exposure demarcation bins are identified for both the magnitudes and durations of the exposure measure [42,43]. The main problem with an approach using EVA is the complexity of the data analysis, since a single analysis might have four exposure levels and seven time sequences, leading to 28 dependent variables [25].

The Shapiro-Wilk test for normality was run on all of the parameters and some were found to violate the assumptions of normality. However, the three parameters just discussed, activity counts per hour, jerk time, and percent time above 30 degrees, were all normally distributed. For the purposes of direct comparison in tables 1 and 2, we have chosen not to perform any transformations on any of the other variables.

There is conflicting evidence in the literature as to whether or not there are inherent side to side differences in daily arm activity in healthy subjects, with some showing differences [5,44] and others finding no differences [7,15,21,45]. For the present study, rather than focus in on whether or not there is a significant difference, we looked at the reliability and ability to detect differences in activity parameters. Looking at differences with t-tests can actually lead to contradictory information. For example, a paired t-test demonstrated a statistical difference between side for activity counts (p < 0.0001) and no statistical difference for the 10^th ^percentile (p = 0.212). So from this analysis, one might conclude that the later parameter would be more appropriate for side to side comparisons in patients. However, the percent difference between the mean dominant and non-dominant scores are similar for these two parameters: 4% for activity counts and 5% for the 10^th ^percentile. The reason that there is a significant difference for activity counts is that due to low variability, this parameter has far more power to detect small differences. Therefore, activity counts would be more likely to demonstrate a significance difference in patients (see table 2).

One main problems with using accelerometers for measuring arm motion is that they are sensitive to linear acceleration resulting from non-zero angular velocities and accelerations. However, studies conducted in our lab [24] and by others [46,47] have determined that these errors are small and predictable. Other sensors have been developed that combine accelerometers, gyroscopes and magnetometers in order to collect three dimensional kinematics [1,21,48-50]. While these more sophisticated sensors hold promise, they also have limitations, such as being large, requiring cables and data loggers worn on a belt and are either very expensive or not commercially available.

There are also several subject-related limitations to the present study. Although hand dominance was not part of our inclusion/exclusion criteria, only 1 out of 32 of our subjects was left handed, compared to a rate of approximately 10% in the general population [51]. Additionally, data were collected in the summer on college students who were not engaged in full time employment. The most common activities reported by the subjects were: walking, preparing/eating meals, computer work, attending classes, riding a bicycle and doing homework. Finally, we only analyzed 3.5 hours of data for each subject, due to limitations with the recording capacity of the VC and some subjects returning earlier than expected. This short data collect is a potential major limitation to the interpretation of the results and as such, we have recently been working with the manufacturers of the VC and they have modified these units to allow for up to 19 hours of data collection. Future studies are planned to validate the results from the present study during full day data collection in workers, as well as testing subjects with upper extremity pathologies.

## Conclusions

In general, the mean activity levels during daily activities were very similar between sides. Specifically, activity counts per hour, jerk time, and percent time above 30 degrees were found to be the variables most likely to reveal significant difference or changes in both individual patients and groups of patients. The use of ambulatory measurements of upper extremity activity has very broad uses for occupational assessments, musculoskeletal injuries of the shoulder, elbow, wrist and hand as well as neurological pathologies.

## Competing interests

The authors declare that they have no competing interests.

## Authors' contributions

MA coordinated and performed the study, helped analyzed the data and wrote the first draft of the manuscript. TA participated in the study design and data collection. ARK conceived of the study, participated in the study design, performed the final statistical analyses and wrote the final draft of the manuscript. All authors approved the final manuscript.

## Pre-publication history

The pre-publication history for this paper can be accessed here:

http://www.biomedcentral.com/1471-2474/11/168/prepub
